# A novel n-type semiconducting biomaterial

**DOI:** 10.1038/s41598-022-26582-4

**Published:** 2022-12-19

**Authors:** Mikio Fukuhara, Tomonori Yokotsuka, Toshiyuki Hashida, Fumio Ogawa, Tadashi Sakamoto, Mitsuhiro Takeda, Susumu Arai

**Affiliations:** 1 New Industry Creation Hatchery Center, Tohoku University, Sendai, 980-8579 Japan; 2 Fracture and Reliability Research Institute, Graduate School of Engineering, Tohoku University, Sendai, 980-8579 Japan; 3 National Institute of Technology, Sendai College, Natori, 981-1239 Japan; 4 Uniparks, Co. Ltd., Funabashi, 274-0826 Japan

**Keywords:** Biophysics, Nanoscience and technology

## Abstract

There has been no research conducted thus far on the semiconducting behaviour of biomaterials. In this study, we present an *n*-type semiconducting biomaterial composed of amorphous kenaf cellulose fibre (AKCF) paper with a voltage-controlled N-type negative resistance. The AKCF generates an alternating-current wave with a frequency of 40.6 MHz from a direct-current voltage source at its threshold voltage (electric field of 5.26 kV/m), which is accompanied by a switching effect with a four-order resistance change at 293 K. This effect is attributed to the voltage-induced occurrence of strong field domains (electric double layers) at the cathode and depletion at the anode of the AKCF device. The proposed AKCF material presents considerable potential for applications in flexible/paper electronic devices such as high frequency power sources and switching effect devices.

## Introduction

Kenaf, or Hibiscus cannabinus, is a plant in the Malvaceae family, which is used to produce rope, twine, coarse cloth, and paper products^1^. In recent years, research has been conducted on this plant since it contributes to the decarbonisation of air^2^ and nitrogen removal from wastewater^3^. Its biodegradable cellulose nanofibers (CNFs) exhibit excellent material and biomedical properties of thermally stability, high durability, and low weight, due to which it has gained significant attention^4–7^. However, there have been no studies conducted on the analysis of the electrical behaviour of amorphous kenaf cellulose fibre (AKCF). We have recently reported that TEMPO-oxidised-amorphous cellulose nanofibers (ACF) supercapacitors with a band gap of 4.77 eV ^8,9^ can store electricity, thereby facilitating the illumination of white LED light and capture of positive and negative charges from air. We have also reported that the ACF is a metastable-like ceramic with isotropic elasticity^10^. A semiconductor composed of renewable and biodegradable paper would be highly advantageous. In this study, we report the generation an alternating current (*AC*) from a direct-current (*DC*) voltage source accompanied by a switching effect with a four-order resistance change in the bulk AKCF, which exhibits a voltage-controlled *n*-type negative resistance at 278 K. In our previous studies^11,12^, we presented a room-temperature amorphous alloy (Ni_0.36_Nb_0.24_Zr_0.40_)_90_H_10_ (bulk) field-effect transistor based on a DC current-induced Coulomb oscillation, which exhibits particle and wave electronic transport^13^. Conversely, in this study, we employ thyristors, which are characterised by *p*–*n* junction semiconductors with switching effect and *n*-type bulk semiconductor Gunn diodes, represented by a harmful compound, GaAs; These diodes function as DC/AC conversion devices with a negative resistance and convert the energy of a constant-voltage power source into high-frequency oscillations owing to the formation of a strong field domain over a certain threshold voltage^14^.

## Results and discussion

### Conversion of DC to AC by AKCF

**Figure 1:**
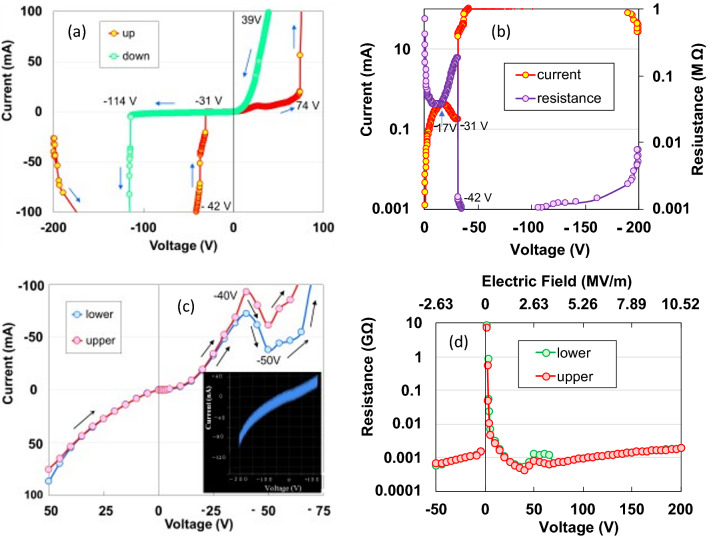
(**a)** *I–V* and *(b)* *R–V* characteristics from − 100 V and 0 V to + 200 V during the up and down runs at a sweep rate of 1.24 V/s, respectively. (**c**) *I–V* characteristics from − 75 to + 50 V, and (**d**) *R–V* characteristics from − 50 to + 200 V under constant voltages. Inset of Fig. 1c shows a curve with an oscillating mode.

We used the DC measuring method to measure the voltage-controlled *I–V* characteristics of the AKCF in the current region from 0 to 100 mA and in the voltage region from − 200 V to + 100 V at 293 K (Fig. 1a). The *I–V* curves indicate nonlinear electron transport behaviour in a metal–semiconductor Schottky junction^15^. They exhibit a radically negative increase from − 200 V, rapid-ohmic decrease down to − 31 V, and an abrupt positive jump at 74 V in the up run, whereas in the down run, they exhibit an abrupt decrease from 39 V, a gradual decrease from − 1 V to − 114 V, and a negative jump from − 114 V. The radically negative increase from − 200 V indicates a N-type negative resistance. The negative resistance characteristics can be classified into static negative resistance characteristics, which account for the of *p- n* junctions in the form of tunnel diodes and thyristors, and dynamic negative resistance characteristics, which account for the carrier travel time and peculiarities of the band structure of the material in the form of impact avalanche transit diodes and Gunn diodes^16^. The hysteresis curves in the up and down runs indicate some undue charge accumulation issues in the experiment. Figure 1b presents the *R–V* characteristic on a logarithmic scale from − 200 and 0 V in both the runs. The *R–V* curve exhibits a three-fold change in magnitude between 0 V and − 42 V, indicating a switching effect. However, the AKCF is a bulk semiconductor (see Supplementary Information (SI) Fig. S6). Figure S4 presents another example of a similar N-type negative resistance. In the *I-V* characteristic of CNF up to 5 runs^9^, however, the current increases with an increase in the voltage and tends to saturate above 4 runs, due to which we cannot repeat the experiment in the high-voltage region near 200 V owing to the 0.1 mA current limitation of our device.

**Figure 2:**
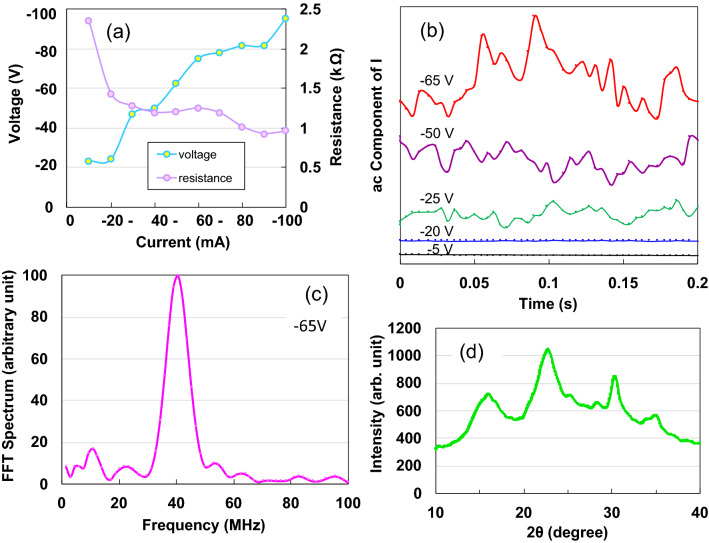
(**a)** *V-I* and *R-I* characteristics from − 100 mA to 0 mA; (**b)** AC voltage component across *R*_*std*_ for various V values; (**c)** FFT spectrum at 65 V; (**d)** XRD analysis results of AKCF specimen.

We measured the current behaviour under a constant voltage from 50 V to − 75 V because the *I–V* curves exhibit hysteresis and the *n*-type semiconductors predominantly exhibit reverse bias *I–V* behaviour. The *I–V* curves (Fig. 1c) show a symmetric increase with respect to zero bias within ± 30 V and further increase with an oscillation mode (inset of Fig. 1c) over − 30 V. Specifically, the curves exhibit differential negative resistance from peak points at − 40 K to valley points at − 50 V. Despite the existence of high- and low-current density regions within a region of negative resistance, the electrical characteristics of the high-current–density regions corresponding to − 70 V could not be measured since the current was limited to 100 mA. Figure 1d depicts logarithmic resistances as a function of voltage. The resistance reduces hyperbolically by four times within one region in the voltage region from 0 to − 40 V. A high electric field of − 5. 26 kV/m is applied at − 40 V; consequently, this can be considered as the material switching from an insulator into an extremely good conductor. Sawano et al.^17^ reported a current-controlled S-type negative resistance-thyristor effect, which functions as a DC–AC (40 Hz) inverter in the conducting organic crystal, *θ*-(BEDT-TTF)_2_CsCo(SCN)_4_ along *b*-axis at 4.2 K. We measured the current behaviour under a constant current condition to exclude the possibility that our device was exhibiting similar behaviour. With the increase in constant current, the voltage increases spontaneously, while the resistance decreases, excluding the current-controlled S-type negative resistance effect, as seen in Fig. 2a^17^.

We measured the AC responses for voltage variation from − 5 to − 65 V, as shown in Fig. 2b, to determine the cause of the voltage oscillations described in the inset of Fig. 1c. Figure 2c depicts the representative FFT spectrum (− 65 V) of the irregular waves, indicating an AC saw wave with voltage oscillation of 40.6 MHz.

### Determining structural morphology and surface characteristics by TEM and AFM

**Figure 3:**
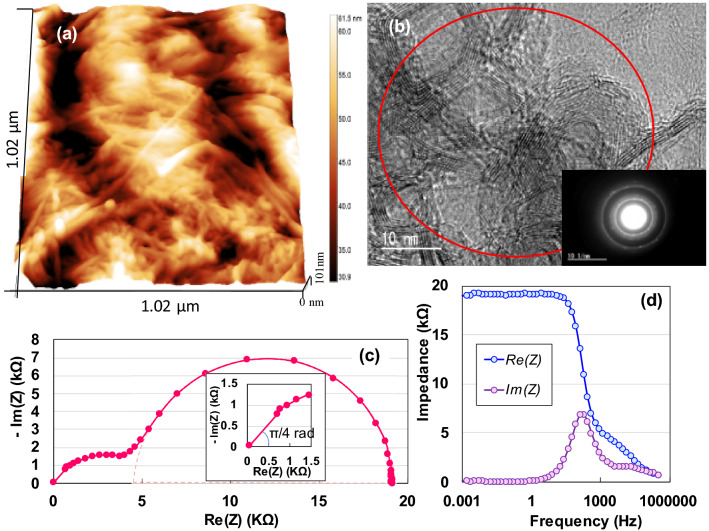
(**a)** AFM and TEM images (**b**) of AKCF specimen and (**b)** SAED pattern of red circle area (inset in (**b**)); (**c**) Nyquist plot as a function of frequency for the AKCF device; (**d**) Real and imaginary impedances.

In this section, we present the analysis of the structural morphologies and surface characteristics of the AKCF specimen. The wide-field X-ray diffraction pattern (Fig. 2d) demonstrates that the specimen comprises an amorphous cellulose phase, whose characteristics are presented by four board peaks at approximately 16°, 23°, 30°, and 35°^18^. Figure 3a presents an atomic force microscopy (AFM) image of the surface structure of the specimen, depicting the features with diameter of 11*–*27 nm (SI S3). The stalk of the Kenaf plant is composed of two types of fibre: an outer fibre (bast) and an inner fibre (core). The bast is comparable to softwood tree fibres such as those of needle-leaf trees (conifers), while the core is comparable to hardwood tree fibres such as those of broadleaf trees^19^. Thus, the sample used in this study might have been composed of two tissues. Figure 3b presents a TEM image of the specimen observed at 120 keV. Cellulose bundles of approximately 4-nm diameter are tied up with nanofibrils of 0.36-nm diameter (SI S2). The inset of Fig. 3b depicts the selected-area electron diffraction (SAED) pattern for irregular line-up nanofibrils obtained from the entire field, indicating diffuse spots and hallow Debye rings in the SAED patterns. Therefore, we consider the irregular nanofibrils are a nanocrystalline phase with a small degree of crystallinity.

### Complex evaluation of semiconducting behaviours

**Figure 4:**
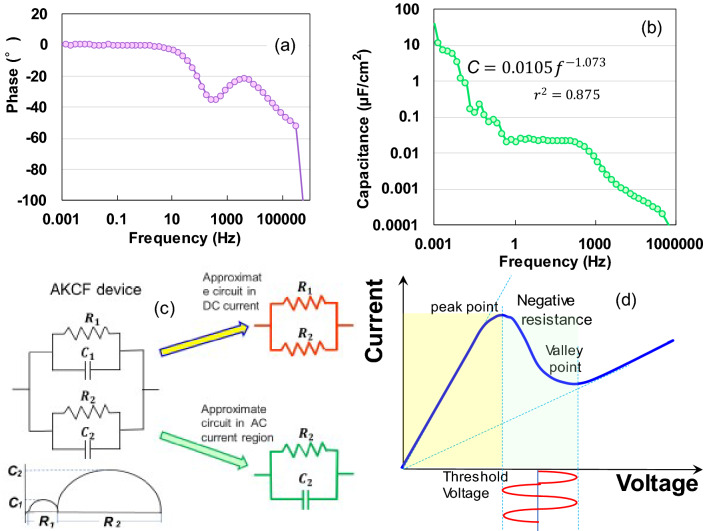
Frequency dependence of (**a**) phase angle and (**b**) parallel capacitance; (**c**) equivalent circuit corresponding to the Nyquist diagram in Fig. 3c and approximate circuits in DC and AC current regions; (**d**) schematic diagram of *I–V* characteristics estimated as Gunn diode.

We measured the AC impedance from 1 mHz to 1 MHz at 300 K to non-destructively analyse the electrostatic contribution of the specimen. Figure 3c presents a complex-plane (Nyquist) plot of the impedance data. The impedance variation of the AKCF with frequency follows the combined pattern of a line slope of π/4 rad and two semicircles with lower and higher resistances. The π/4 − rad region (Warburg regions) depicted in the inset of Fig. 3c is attributed to the distributed resistance/capacitance in a porous electrode^20,21^. The two semicircles may represent the tissue composed of fibres from the bast and core, as can be observed from the AFM image in Fig. 3a. The large semicircle indicates that the electrode is a kenaf film with a porous surface with a high resistance. The second semicircle is a true semicircle, based on the Debye-type relaxation model. The relaxation time of 0.001 s can be calculated using *RC*_*total*_ = 1/(2*πf*_*max*_), where *f*_*max*_ (= 158.9 Hz) represents the peak frequency of the second semicircle, indicating interfacial polarisation in the audio frequency range^22^. Figure 3d depicts the frequency dependence (Bode diagram) of the real and imaginary impedances. We can observe a rapid increase in the middle-frequency region followed by saturation of the real impedance below11.9 Hz and a single peak in the imaginary impedance at 158.9 Hz, indicating a dielectric dispersion caused by interfacial polarisation. Furthermore, the capacitive behaviour (near the zero phase angle) in the frequency region from 0.02 Hz (Fig. 4a) clearly demonstrates a parallel-*RC* circuit. The parallel capacitance, *C*_*p*_, was obtained as 39 μF/cm^2^ at 1 mHz (Fig. 4b). Since *C*_*p*_ = 0.019f ^− 1.073^ (*r*^*2*^ = 0.875) for 1 mHz < *f* < 1 Hz, the DC capacitance can be significantly increased (see SI Fig. S6), even if it cannot be observed.

Lastly, we consider the origin of the effect which converts DC to AC in AKCF. Figure 4c presents an equivalent parallel circuit with two semicircles corresponding to the Nyquist diagram in Fig. 3c. The transport resistances are *R*_*1*_ = 5.82 kΩ and *R*_*2*_ = 19.2 kΩ, and the electric double layer capacitances are *C*_*1*_ = 2.02 × 10^–9^ F and *C*_*2*_ = 5.22 × 10^–8^ F (SI S6). These behaviours resemble those of a Gunn diode, except for the higher frequencies, since the AKCF is characterised by the voltage-controlled *N*-type negative resistance effect, By analogy with the Ridley*–*Watkins*–*Hilsum mechanism^23–25^, we infer that the electrons in the lower valley (Fig S7) of the conduction band with smaller *R*_*1*_ move into the higher valley with larger *R*_*2*_ where their mobility decreases due to an increase in their effective mass at a threshold voltage of 5.26 kV/m at 40 V (yellow region in Fig. 4d). This reduction in mobility decreases the conductivity (green region in Fig. 4d). The AKCF can be considered as a solid electrolyte in the semiconductor group owing to its resistivity, which is calculated as 3.45 × 10^4^ Ωm (SI S9, Fig. S7). The homogeneous charge and electric field distribution are unstable at a negative differential resistance, presenting the possibility of domain formation. Therefore, the Gunn effect is estimated by the fact that a dipole domain (electric double layer^25^) with *C*_*2*_ periodically arises at cathodic electrode under a strong electric field, which then moves and depletes at the anodic electrode. The amorphous phase with several atomic defects effectively forms strong electric field regions (domains) due to the electric field concentration. The protonic soliton contributes to generation of large electrical charges by forming a pair of particles comprising an electron and a proton^9^. Therefore, the Debye-type dielectric relaxation and DC conductivity (Fig. 4b) are attributed to the dynamics of a collective proton-relay along the hybrid hydrogen-bonded one-dimensional chain^26,27^. The switching and DC/AC conversion effects must be analysed further. Further studies will be focused on the electric field dependence of the electron mobility in the material using the Hall effect measurements and the calculated energy band structure of the material with donor levels due to mineral impurities. Furthermore, the semiconducting properties of Kenaf need to be compared with those of woods such as softwoods and hardwoods.

## Conclusion

In this study, we analysed the electric and dielectric properties of an AKCF. The sample exhibited a voltage-controlled N-type negative resistance under DC and Debye-type relaxation under AC at 158.9 Hz. Our results demonstrate that the AKCF can be implemented as a novel biomaterial semiconductor diode, which can be applied in carbon–neutral paper electronic devices.

## Methods

The AKCF specimen was fabricated on an Si substrate through spin coating, which was performed at a speed of 400 rpm for 5 s, using 2.6% (w/v) AKCF/water dispersion. The AKCF films were dried in a ventilated oven at 373 K. The specimens (12 mm wide, 19 μm thick, and 15 mm long) were mechanically sandwiched by two Al electrodes. The sample structure was examined by performing XRD, SAED, and AFM analyses. The current–voltage (*I–V*) and resistivity-voltage (*R–V*) characteristics were measured under DC voltages from − 200 to 200 V in air at a sweep rate of 1.24 V/s using a Precision Source/Measure Unit (B2911A, Agilent). The AC impedance and frequency were measured using a potentiostat/galvanostat (SP-150, BioLogic Science) and a mixed-signal oscilloscope (MSO 5104), respectively.

## Supplementary Information

Supplementary Information.

## Data Availability

The data that support the findings of this study are available within this article and its Supplementary Information. Additional data are available from the corresponding authors on request.
