# Supplementary material for: A novel n-type semiconducting biomaterial
Source: Sci Rep. 2022 Dec 19;12:21899. doi: 10.1038/s41598-022-26582-4 (PMC9763477; doi:10.1038/s41598-022-26582-4)
Supplement: Supplementary file 1 — Supplementary Information. [file 41598_2022_26582_MOESM1_ESM.pdf]

SUPPLEMENTARY INFORMATION

A novel *n*-type semiconducting biomaterial

Mikio Fukuhara,[1] Tomonori Yokotsuka,[1] Toshiyuki Hashida,[2] Fumio Ogawa,[2] Tadashi Sakamoto,[2] Mitsuhiro Takeda[3] and Susumu Arai[4]

[1] New Industry Creation Hatchery Center, Tohoku University, Sendai 980-8579, Japan,

[2] Fracture and Reliability Research Institute, Graduate School of Engineering, Tohoku University, Sendai 980-8579, Japan,

[3] National Institute of Technology, Sendai College, Natori, 981-1239, Japan

[4] Uniparks, Co. Ltd., Funabashi 274-0826, Japan,

S1. Methods

Dried bast kenaf pulp fibres (harvested in Bangladesh, Toho Tokushu Pulp Corp. Kitakami, Japan) were retted in a 2.6 % water solution for 18 ks. They were then defibrated in a mixer for 1.2 s. Subsequently, they were miniaturized through ball milling using zirconia balls for 36 ks. The sample structure was analysed through X-ray diffraction (XRD) in the reflection mode with monochromatic Cu Kα radiation. A selected-area electron diffraction (SAED) analysis was performed using transmission electron microscopy (JEM-2100, JEOL). The surface morphologies were analysed via atomic force microscopy (NanoScope V/Dimension Icon, Bruker AXS). All electronic measurements were performed in an Al shield box to present the results from being affected by the electromagnetic interference from surroundings. Fig. S1 presents a schematic diagram of the experimental circuit.

S2. TEM image of AKCF

The cellulose structure is characterised by a mixed structure, which primarily comprises irregular lined-up nanofibrils along with a small quantity of non-fibrous fibrils. The cellulose bundles are tied up with nanofibrils with a diameter of 0.36 nm to form a cellulose nanofiber (CNF) with a diameter of approximately 4 nm.

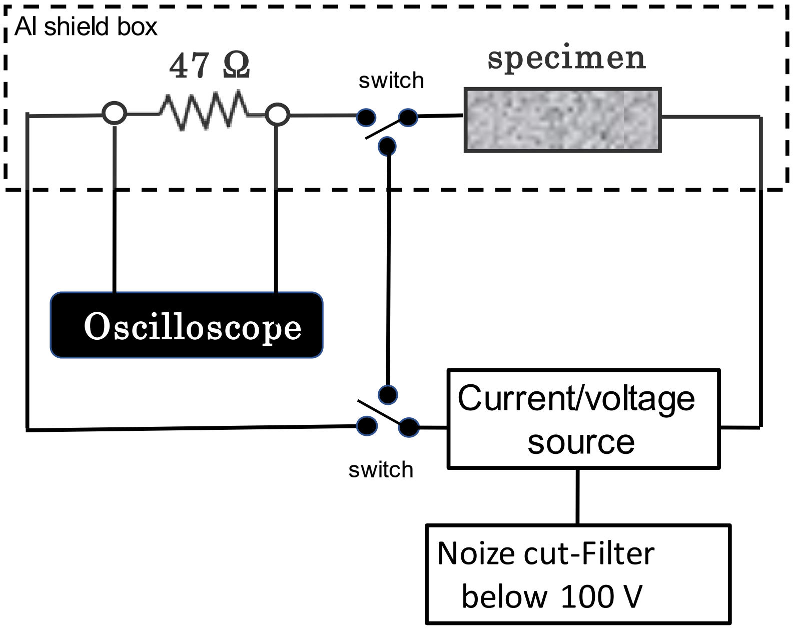

Fig. S1 Experimental circuit schematic diagram.

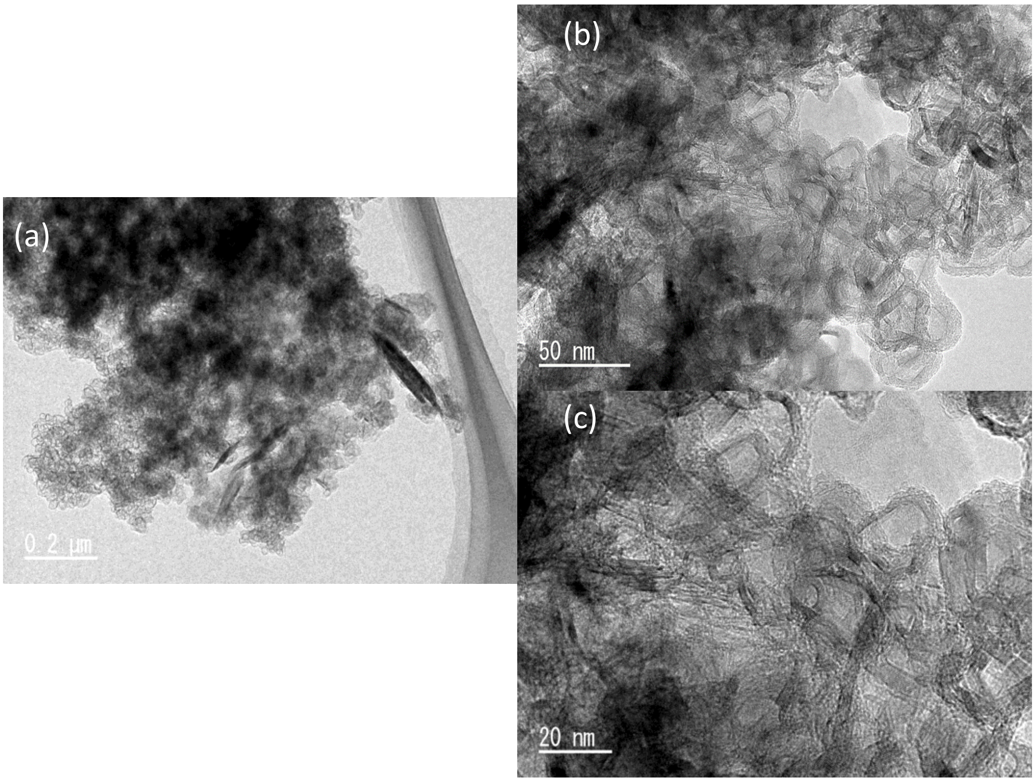

Fig. S2 Transmission electron microscope (TEM) images of AKCF specimen observed at 120 keV.

S3. AFM image of AKCF

Figure S3(a) presents the atomic force microscope (AFM) image of the AKCF sample. Profiles 1 (b) and 2 (c) depict the heights measured from the valley along the black lines in the AFM image (a), revealing convex distances of 10.6 and 27.0 nm, respectively. The diameters are three to five times larger than that of AKCF, which is ~4 nm, as shown in Fig. 3b. This could be attributed to the condensation of cellulose bundles due to strong thixotropy of the AKCF. Thixotropy is an effect in which the viscosity depends on both the velocity gradient as well as the time for which force has been applied[28]. The faster a thixotropic liquid moves, the less viscus it becomes.

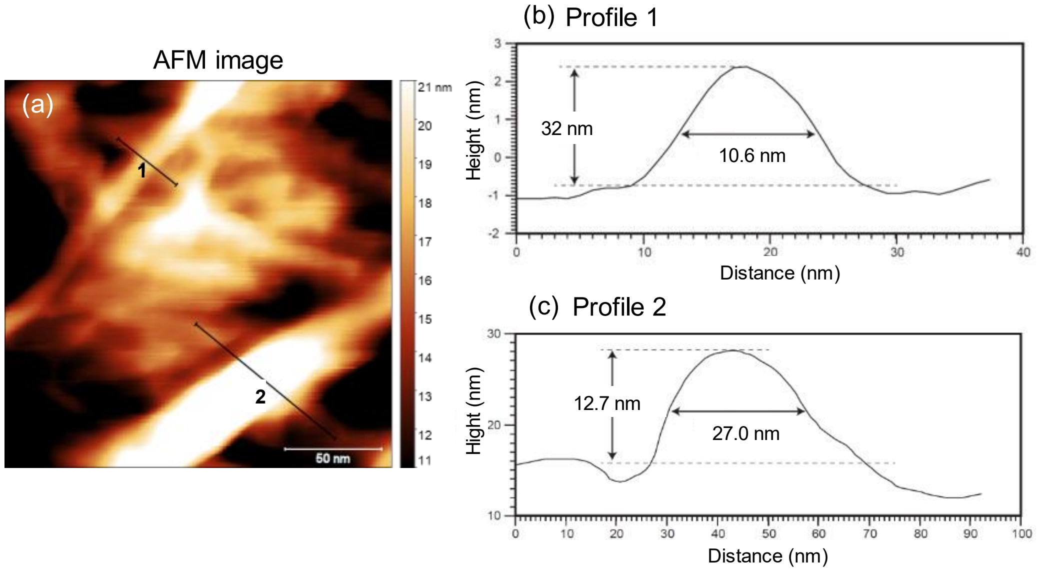

Fig. S3 (a) AFM image. (b) Upper and (c) lower profiles are the heights measured from the valley along the red lines in the AFM image.

S4. Repeatability of *IV* sweep

Figure S4 presents another repeated result of the *I*-*V* and *R*-*V* characteristics of an amorphous cellulose nanofiber (ACF) with a thickness of 118 μm, to provide a better understanding of the cyclic behaviour observed in this study. It depicts the same *N*-type negative resistance and a switching effect with a three-order resistance change as those in

in Figs. 1(a) and (b), respectively.

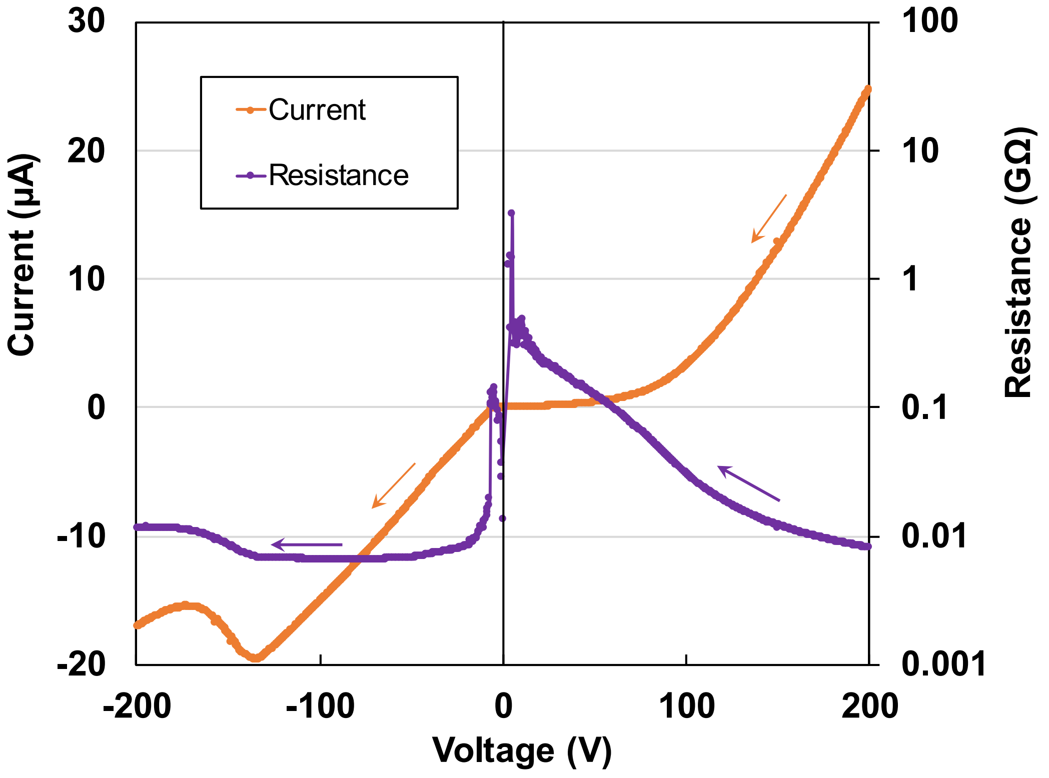

Fig. S4 *I–V* and *R–V* characteristics from ＋200 V to -200 V during down run at a sweep rate of 1.24 V/s.

S5. The calculated energy band structure of AKCF

The molecular structure of cellulose of both kenaf and softwood are identical. Consequently, the density of states for the $C_{12}H_{20}O_{10}$ molecule optimized from the local structure of cellulose was simulated. Figure S5 depicts a band gap of - 4.77 eV [8,9].

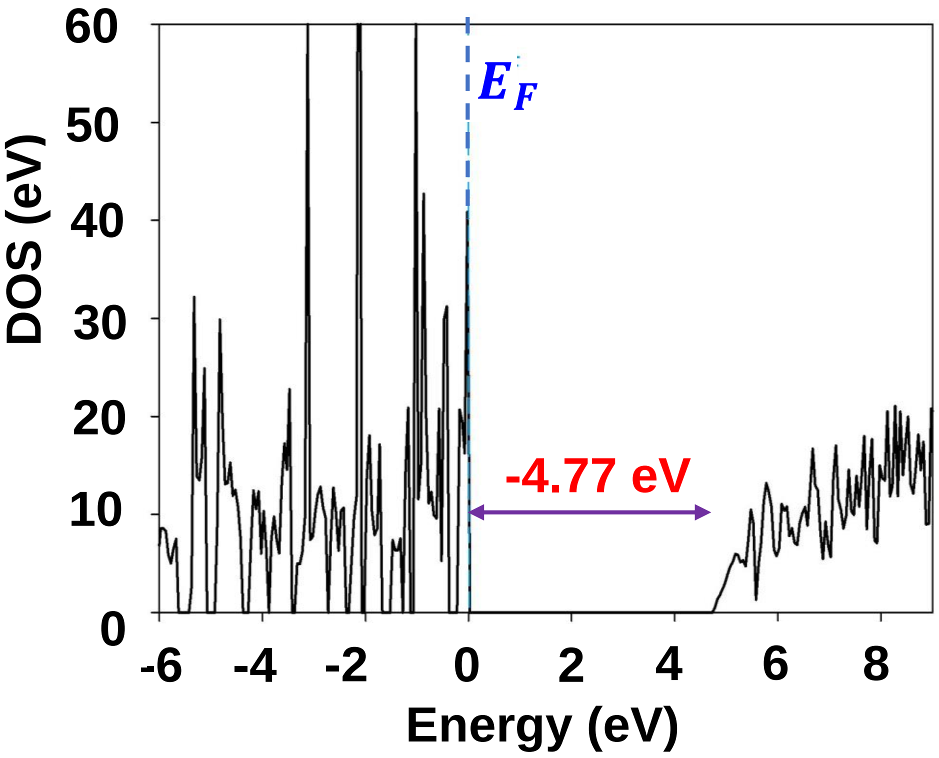

Fig. S5 Density of states (DOS) in $C_{12}H_{20}O_{10}$.

S6. Calculation of resistances and capacitances

We calculated the resistances, R_1 and R_2, and capacitances, C_1 and C_2, based on the small and large semicircles in Fig. 3c, respectively. The maximum frequencies for the small and large circles are 13,554.7 Hz and 158.9 Hz, and their maximum resistances are 3.64 (3.45 × 10^4 Ωm = 3.64 kΩ × 1.2 cm × 1.5 cm / 0.0019 cm) and 19.2 kΩ (1.82 × 10^5 Ωm = 19.2 kΩ × 1.2 cm × 1.5 cm / 0.0019 cm), respectively. Thus, the capacitances can be calculated from the formula C_{total}= $1/(2\pi f_{max})$ R; C_1 = 3.23 ×10^{-9} and C_2 = 5.22 ×10^{-8} F. We then obtained R_1C_1 = 1.174 × 10^{-5} s and R_2C_2 = 1.002 × 10^{-3} s. Subsequently, we calculated the capacitance Cs from Cs = $0.019f^{-1.073}$ (Fig. 4b). Figure S6 depicts the estimated capacitance as a function of frequency (f) below 100 mHz.

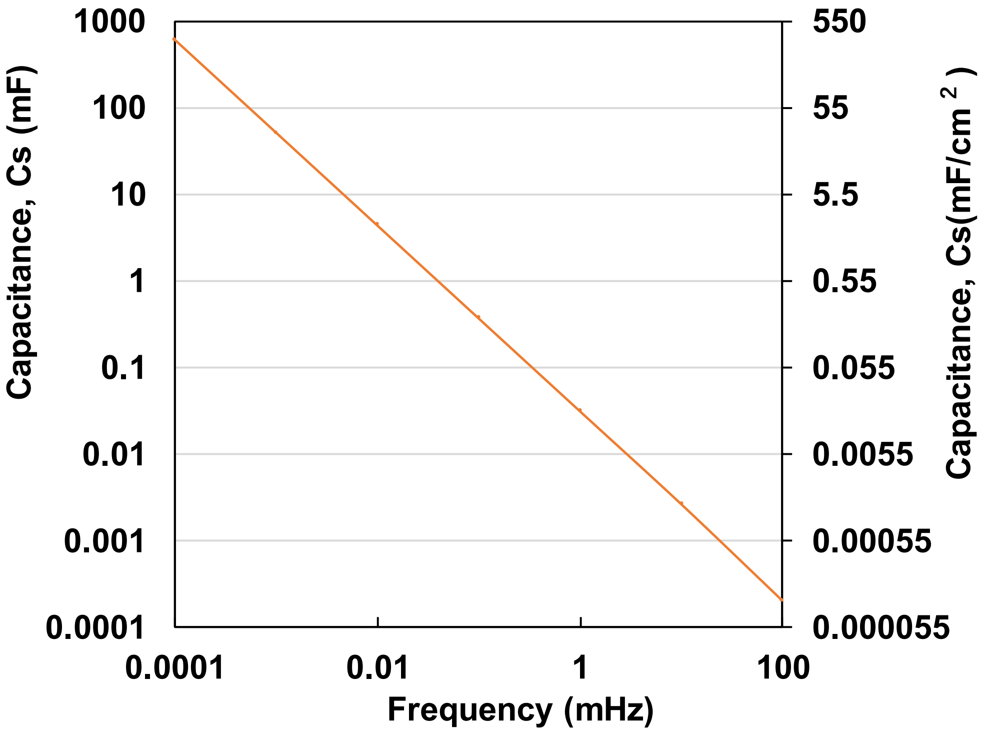

Fig. S6 Calculated capacitance from Cs = $0.019f^{-1.073}$ as a function of frequency below 100 mHz.

S7. Correlation between dielectric response and DC conductivity

Tokura *et al.* [29] reported a significant experimental correlation between dielectric response such as dielectric relaxation and DC conductivity for various organic ionic donor–acceptor charge-transfer compounds. They observed through an experimental analysis that kink-type domain walls exist between one-dimensional ferroelectric molecular domains. In this study, we observed ideal dielectric relaxation (Fig. 3d) and large DC conductivity (Fig. 1d) in the AKCF biomaterial. Based on these results, we concluded that dielectric domains are transferred from the cathodic electrode to anodic electrodes by voltages which are greater the threshold voltage (electric field of 5.26 kV/m). The domain is the electric double layer in the solid electrolyte. The pairs of oppositely charged species composed of electrons and protons account for the unusually large dielectric response produced when they are bound and for the charge transport when they are dissociated[30]. Kashimori *et al.*[31] discussed the solitonic mechanism for proton transport in hydrogen bonded chains in biological systems.

Figure 1a depicts an abrupt increase in current at -114 and 74 V. However, this is not considered as an electron avalanche, since the AKCF is not a diode that comprises a *p-n* junction but a passive semiconductor device with two terminals comprising only *n*-type semiconductor material.

S8. Depth dependence of frequency

The frequencies were determined by samples of varying thickness, not by an external circuit. We observed a correlation between the frequency and depth of specimens (Fig.

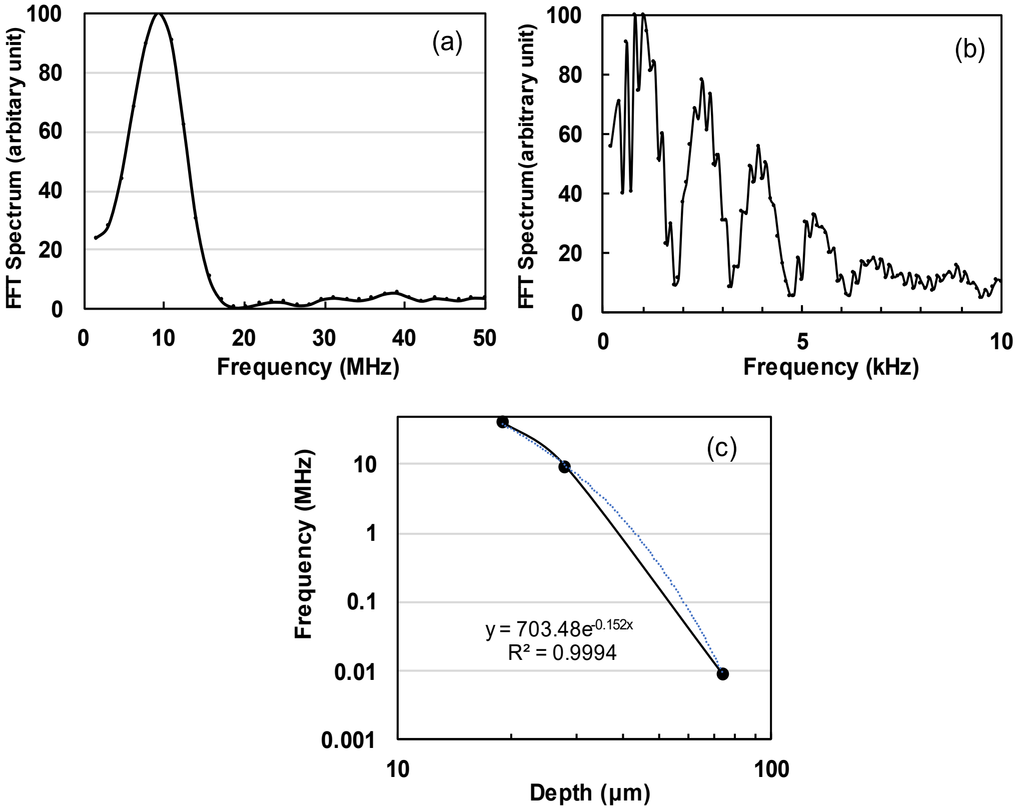

Fig. S7 FFT spectra of 28 μm (a) and 74 μm (b) of specimens. (c) Depth dependence of frequency.

S7c) based on the following values: 60.4 MHz at 19 μm (Fig. 2c), 9.4 MHz at 28 μm (Fig. S6a), and 9 kHz at 74 μm (Fig. S7b). Figure S7b depicts the sixth-order harmonic AC waves with frequencies of 1 kHz, 2.4 kHz, 3.8 kHz, 5.2 kHz, 6.6 kHz, and 9 kHz. However, the reason behind the generation of these harmonics remains unclear.

S9. Transition of electrons in bands

We observed two semicircles in the Nyquist diagram presented in Fig. 3c. It can be observed from Fig. S8 that the electric resistivities obtained from the small and large semicircles belong to the semiconductor group.

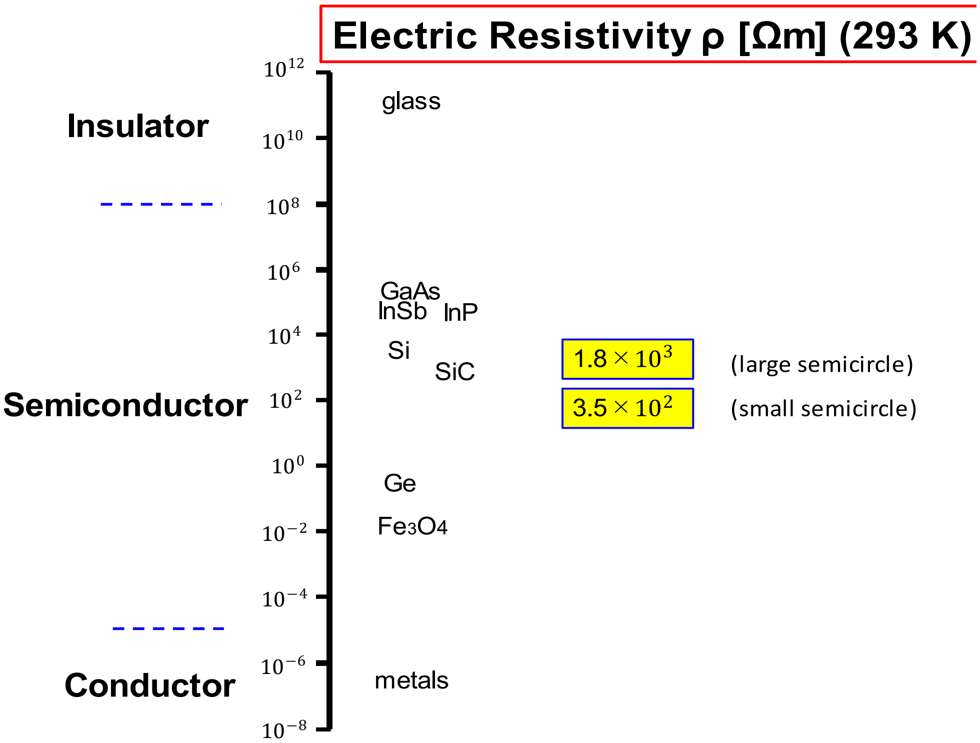

Fig. S8 Electric resistivity of the two semicircles in Fig. 3c

A Gunn diode is characterised by electron transfer between a lower band and a higher band[22-24], as shown in Fig. S9. Therefore, we must consider the electron mass and drift velocity in the upper band. Firstly, we consider the electron mass. Since the electric dipole domain is formed by an electron and proton pair in a CNF, the drift velocity of the pair is determined by the heavier mass (1.67×10^{-24} g) of the proton. The mass ratio of m_2 in the upper band to m_1 in the lower band is 1836: 14 ($1.67 \times 10^{-24} + 9.11 \times 10^{-28}$) g /$9.11 \times 10^{-28}$ g), such that $m_2 = 1836.14\ m_1$.

Gunn diode is based on the transport of a strong electric field region (domain), and its period of modulated current depends on the travel time of dipole domains. Thus, the ratio of m_2/m_1 and R_2C_2 constant of the upper band conveniently serve as a measure of electric capability and the time constant in place of the effective mass and drift time, respectively. We obtain μ_2 = 7.72 10^2m/s (= l /T = $f\,l$ =4.06×10^7 (1/s) $\times 1.9 \times 10^{-5}$ m) at frequency f = 40.6 MHz, where l and μ_2 present the distance between the cathodic and anodic

electrodes and draft velocity of the upper valley, respectively. Thus, we obtain μ_1 = 1.39×10^6m/s from $m_1\mu_1 = m_2\mu_2$.

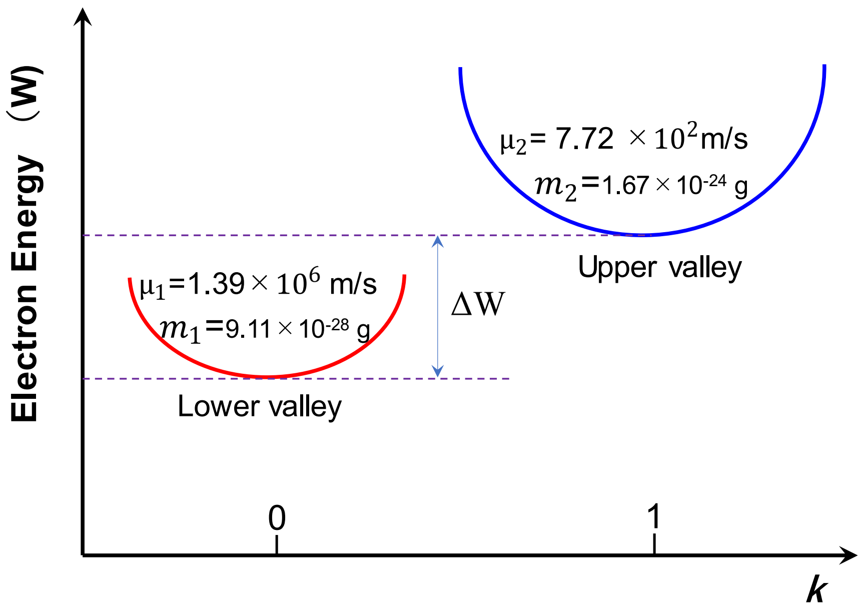

Fig. S9 Zone structure of AKCF.

References

[28] Dictionary Physics, Wagner Communications Company, New York, 1985, p. 269.

[29] Tokura, Y, et al., Domain-wall dynamics in organic charge-transfer compounds with one-dimensional ferroelectricity, *Phys. Rev. Lett.*, **63**, 2405 – 2408 (1989).

[30] Sugawara, T., Terao, H. & Takasu, I., Proton dynamics in hydrogen-bonded molecular crystals, *J. Cryst. Soc. Jpn*. **43**, 13–20 (2001).

[31.] Kashimori, Y., Kikuchi, T. & Nishimoto, K., The solitonic mechanism for proton transport in a hydrogen bonded chain, *J. Chem. Phys*. **77**, 1904–1907 (1982).
